# Preventive treatment with CGRP monoclonal antibodies restores brain stem habituation deficits and excitability to painful stimuli in migraine: results from a prospective case-control study

**DOI:** 10.1186/s10194-021-01364-x

**Published:** 2021-12-11

**Authors:** Anne Thiele, Lara Klehr, Sebastian Strauß, Anselm Angermaier, Ulf Schminke, Martin Kronenbuerger, Steffen Naegel, Robert Fleischmann

**Affiliations:** 1 Department of Neurology, University Medicine Greifswald, Ferdinand-Sauerbruch-Str. 1, 17475 Greifswald, Germany; 2 University Hospital of Old Age Psychiatry and Psychotherapy, University of Bern, Bern, Switzerland; 3 Department of Neurology, Martin Luther University Halle- Wittenberg and University Hospital Halle, Halle (Saale), Germany

**Keywords:** Migraine, Headache, Prevention, Calcitonin gene-related peptide, Antibodies, Disease modifying drug, Blink reflex

## Abstract

**Background & Objectives:**

Calcitonin gene-related peptide ligand/receptor (CGRP) antibodies effectively reduce headache frequency in migraine. It is understood that they act peripherally, which raises the question whether treatment merely interferes with the last stage of headache generation or, alternatively, causes secondary adaptations in the central nervous system and might thus possess disease modifying potential. This study addresses this question by investigating the nociceptive blink reflex (nBR), which is closely tied to central disease activity, before and after treatment with CGRP antibodies.

**Methods:**

We enrolled 22 patients suffering episodic migraine (21 female, 46.2 ± 13.8 years of age) and 22 age-/gender-matched controls. Patients received assessments of the nBR (R2 component, 10 trials, 6 stimuli/trial) before (V0) and three months (V3) after treatment with CGRP antibodies started, controls were assessed once. The R2 area (R2a) and habituation (R2h; gradient of R2a against stimulus order) of the stimulated/non-stimulated side (_s/_ns) following repeated supraorbital stimulation provide a direct readout of brainstem excitability and habituation as key mechanisms in migraine.

**Results:**

All patients showed a substantial reduction of headache days/month (V0: 12.4±3.3, V3: 6.6 ± 4.9). R2a_s (F_global_=5.86, *p*<0.001; block 1: R2a_s: -28%, *p*<0.001) and R2a_ns (F_global_=8.22, *p*<0.001, block 1: R2a_ns: -22%, *p*=0.003) were significantly decreased, and R2h_ns was significantly enhanced (F_global_=3.07, *p*<0.001; block 6: R2h_ns: *r*=-1.36, *p*=0.007) from V0 to V3. The global test for changes of R2h_s was non-significant (F_global_=1.46, *p*=0.095). Changes of R2h significantly correlated with improvement of headache frequency (R2h_s, *r*=0.56, *p*=0.010; R2h_ns: *r*=0.45, *p*=0.045). None of the nBR parameters assessed at baseline predicted treatment response.

**Discussion:**

We provide evidence that three months of treatment with CGRP antibodies restores brain stem responses to painful stimuli and thus might be considered disease modifying. The nociceptive blink reflex may provide a biomarker to monitor central disease activity. Future studies should evaluate the blink reflex as a clinical biomarker to predict treatment response at baseline and to establish the risk of relapse after treatment discontinuation.

**Trial registration:**

This trial was prospectively registered at clinicaltrials.gov (ID: NCT04019496, date of registration: July 15, 2019).

## Background

Migraine is among the most prevalent neurological disorders and substantially interferes with individuals’ psychosocial health, family life and professional development [1]. An effective preventive treatment is considered key to ameliorate the negative impact of migraine [2]. Unfortunately, most approved preventive drugs are characterized by low tolerability and contraindications that impede their unconditional use in clinical routine [3, 4]. Monoclonal antibodies targeting calcitonin gene-related peptide (CGRP) or the CGRP receptor (hereafter collectively referred to as *CGRP mAbs*) have ushered in a novel era of migraine prevention by being specifically designed to target disease relevant mechanisms, which enables superior tolerability and efficacy to oral preventives [5, 6].

Some practical issues in clinical routine application of CGRP mAbs remain, importantly patient selection and duration of treatment, which are both essential to be resolved given associated treatment costs [7]. German guidelines recommend continuing treatment for about six to nine months if they were proven effective in a preceding three-month trial [3]. European guidelines are less absolute about the treatment duration but also advise to continue for about 6-12 months while the American Headache Society Consensus Statement considers reauthorization duration indefinite and should be guided by patient response and medical professional attestation [8, 9]. This discrepancy of guidelines reveals uncertainty about long-term effects of CGRP mAbs on the natural course of migraine, but it is generally acknowledged that the preventive treatment should potentially lead to a sustained amelioration of headache frequency as it is shown for oral preventives, which may be considered disease modifying [10–12]. While there is no unequivocal definition of disease modification in headache research, it might follow the European Medicines Agency’s definition as “slowing or arrest of symptom progression and evidence of delay in the underlying […] pathophysiological disease processes” [13]. Martelletti proposed in analogy that a Disease-Modifying-Migraine-Drug (DMMD) should “slow down or freeze or revert the natural course of migraine” [14]. Currently, it is unclear if a course of CGRP mAbs possesses disease modifying potential. This concern is mainly due to the presumed peripheral mode of action, which is blocking the effect of CGRP release in the trigeminovascular system and not directly targeting central structures [5, 15, 16]. Furthermore, up to 80% of patients relapse to their prior headache frequency following discontinuation of the treatment with CGRP mAbs, which further challenges central disease modifying potential, but may be confounded by non-pharmacological effects [17, 18]. On the other hand, functional imaging studies revealed altered activity in brain regions such as the hypothalamus closely related to migraine pathophysiology following treatment with CGRP mAbs, which argues for disease modifying activity [19]. The discrepancy of pharmacological, clinical and imaging studies precludes conclusions whether or not long-term treatment with CGRP mAbs shapes the natural course of migraine. Consequently, there is no biomarker to select suitable patients and the ideal moment for discontinuation of treatment with CGRP mAbs.

This study aims to address this ambiguity of findings by investigating patients with migraine treated with CGRP mAbs for longitudinal changes of brain stem excitability and habituation to nociceptive stimuli, which were shown to be deficient in migraine and closely tied to central disease activity [15, 20]. The nociceptive blink reflex (nBR) was repeatedly proven to be an ideal neurophysiologic biomarker for this purpose since it provides a direct readout of central processing of trigeminal sensory (nociceptive) input, and is hence used as the primary endpoint to probe disease modification potential in this study [21–23]. Findings from this study may also yield implications for the nBR as therapeutic biomarker in migraine.

## Methods

### Ethical approval and study registration

This study was prospectively registered at clinicaltrials.gov (Identifier: NCT04019496) and approved by the ethics committee of the University Medicine Greifswald (Identifier: BB 168/18). All procedures adhered to the *Helsinki declaration* in its latest revision and were conducted in line with current guidelines for *good clinical practice* (ICH E6(R2)). All patients and controls were provided detailed study information and gave their written consent for the study and the use of their data.

### Study design and participant selection

This is a prospective case-control study with patients suffering episodic migraine serving as cases and healthy volunteers serving as matched controls. Matching was done for age (±5 years) and gender. Controls were required not to suffer from any neurological condition or a primary headache disorder (defined as headache frequency of <1/year and negative medical history).

Patients suffering migraine were identified among patients presenting to the specialized headache outpatient clinic affiliated with the Department of Neurology of a tertiary care university hospital in Northern Germany. Diagnosis of migraine was established according to international classification of headache disorders, 3rd revision, (ICHD-3) criteria [24]. Only patients with episodic migraine with an indication for preventive treatment with CGRP mAbs were considered. Chronic migraine was an exclusion criterion since at least a proportion of patients lacks a clear interictal phase that was required for BR assessments [25]. Further exclusion criteria for both groups were presence of a medication-overuse headache and chronic intake of central nervous system active drug.

Cases were investigated after washout of any previous preventive drug, defined as five half-lives, at least two days apart from any preceding headache attack, and before starting treatment with CGRP mAbs (visit at zero months of treatment, i.e. usually the day of the first injection, denoted as V0) and three months (±14 days) after treatment was initiated (denoted as V3). The rationale for a three-month interval between visits was treatment response is to be expected and can be assessed within that period [3, 5]. Controls were investigated only once at about the same time of the day.

### Primary endpoint – blink reflex habituation and sensitization

**Fig. 1:**
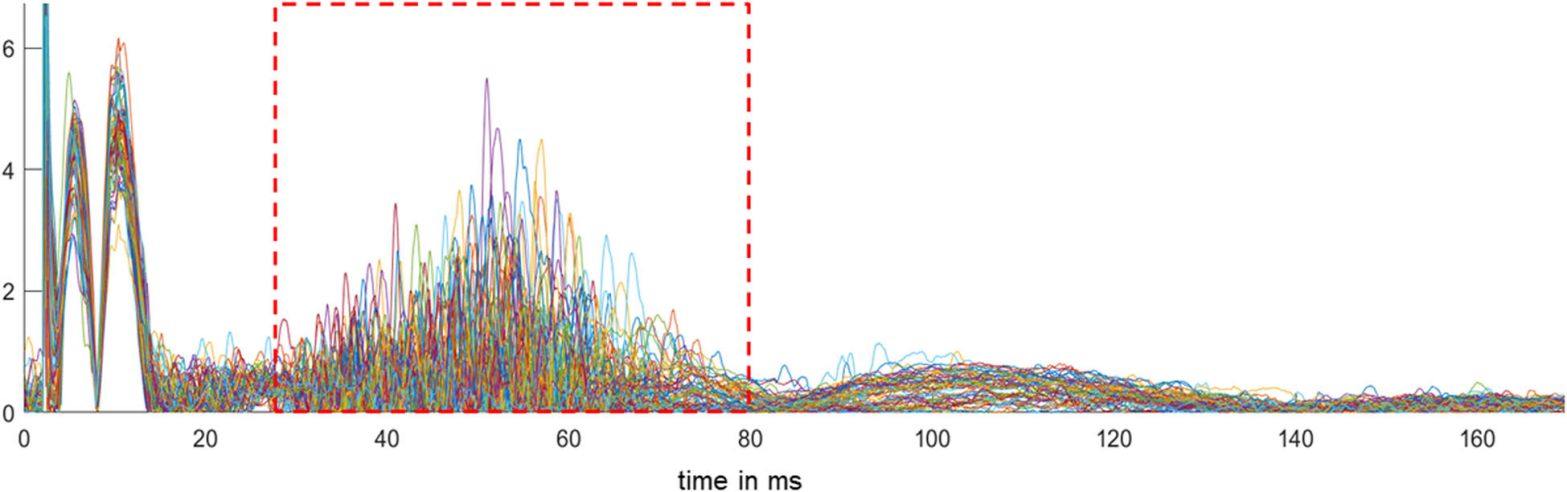
Superimposed 60 nBR traces of one participant. The R2 component is indicated by a box of red dashed lines between 30-80 ms. Note the stimulation artifact at the start and the immediately following R1 component. The R1 component has a low variability given its monosynaptic pathway through the principal trigeminal nucleus. The R2 component is more variable in amplitude and time given its polysynaptic pathway through the trigemino-cervical complex. The R3 follows the R2 component and starts at about 80-90 ms. Its underlying pathways and functions are still debated

The area under the curve (AUC) and habituation of the BR’s R2-component following repeated stimulation were investigated as primary endpoints [26]. The nBR response was elicited and evaluated using well-established electrical stimulation parameters in migraine research in order to enable comparison to previous results [26, 27]. Painful electrical stimuli were applied to the supraorbital division of the trigeminal nerve on the main headache side (matched in controls) through a commercial electrophysiology setup (Neuropack X1, Nihon Kohden Europe, Rosbach, Germany) using a bipolar montage of gold cup electrodes, which were fixed using adhesive paste and tape. We decided to use a classic bipolar montage for two reasons. First, using ring electrodes with a circular anode and central cathode were shown to specifically activate Aδ-fibers, which elicits a nociception specific BR (nsBR), and may be influenced by direct peripheral antagonism of CGRP mAbs at the time of stimulation and confound assessments of central neuroplastic effects [28, 29]. Second, this approach was chosen for ready implementation of findings in electrophysiological laboratories. Surface electromyography was recorded using a bipolar montage with the refence being placed over the tip of the nose and active electrodes being placed over bilateral orbicularis oculi muscles [30]. Electrode positions were recorded and replicated at V3 in patients. Stimulation parameters were set as previously recommended by a panel of experts [26]. In brief, the pain threshold (PT) was established using stimuli, which were at least 30 s apart to avoid habituation, at increasing intensity. Then, 60 stimuli with a pulse width of 0.3 ms and 1.5x PT intensity were applied in 10 blocks each consisting of 6 stimuli with an interstimulus interval (ISI) of 15-17 s and an interblock interval of at least 2 min. Traces of recorded EMG responses were then exported into a MATLAB environment and the R2 component defined as responses that occurred in an interval of 30-80 ms (see Fig. 1).

Preprocessing of CMAPs was done using the fieldtrip toolbox and included rectification, detrending and z-transformation, which was done to enable comparisons of R2 AUC (R2a) intra- and interindividually, and between sides (indicated by suffix: _s = stimulated, _ns = not-stimulated) [31].

### Secondary endpoints

Demographic characteristics were recorded in all participants, which included age and gender. We additionally assessed headache characteristics (headache frequency, mean headache intensity [rated on a numerical scale ranging from 0 to 10 points], attack duration, time the last headache occurred before and after the assessment) and headache-related disability (Migraine Disability Assessment [MIDAS], Headache Impact Test [HIT-6^TM^]) in patients suffering migraine through tools highly recommended by the National Institute of Neurological Disorders and Stroke common data element initiative [32]. All patients were obliged to provide a headache diary starting 3 months before beginning CGRP mAb treatment and throughout the study period.

### Sample size considerations, data evaluation and statistics

The mean effect size of previous studies using the R2 AUC and habituation for the assessment of treatment response found a mean effect size of 0.7 (Cohen’s *d*) [15, 21, 26]. G*Power (v.3.1.9.2, University of Düsseldorf, Germany) was used to calculated the sample size required to detect R2 changes between V0 and V3 in patients using a two-sided paired t-Test with an alpha-error of 0.05, power of 90% [33]. The power calculation revealed that 22 patients, and thus 22 controls, would be required to test our hypothesis. We aimed to include 25 participants in each group to account for about 10% drop-out rate.

NBR data were pre-processed as described above. The AUC of R2 responses (R2a) was computed using a trapezoidal approximation of its integral. Habituation (R2h) was quantified as the beta coefficient (i.e. β0, slope) of the linear regression: f(R2a_i_) = β0*R2a_i_ + intercept (i = stimulus order). A positive slope indicates facilitation, negative slope habituation and zero slope no change of the trigemino-cervical complex (TCC) to consecutive stimulation. Evaluations of R2a and R2h corrected for the time before the following headache attacks were included as exploratory endpoints.

Further statistical analyses were carried out using the Statistical Package for the Social Sciences (SPSS v25.0, IBM, Armonk, NY, USA). Continuous data were analysed for normal distribution using histogram plots before performing descriptive and inferential statistics. Unless stated differently, normal distribution was confirmed. Descriptive normal distributed data are presented as group means ± standard deviation. Inferential comparisons within and between group means were done using repeated-measures analysis for variance (rmANOVA, patients) or ANOVA (patients vs. controls) to test for global effects. If global effects, e.g. difference between visits, were present, single parameters were post-hoc compared pairwise and were corrected for multiple comparisons using the Bonferroni method. Frequencies are reported numerical.

Pearson correlation coefficients were determined to evaluate whether changes in electrophysiological parameters correlated with clinical response. Linear regression was used to evaluate whether electrophysiological parameters at V0 predicted treatment response in terms of headache days. Results of linear and binary regression analyses are presented using beta coefficients or odds ratios including their 95% confidence interval (95%CI), respectively.

P-values below 0.05 were considered significant, results below 0.001 are not reported exact but as 0.001.

## Results

### Demographics and clinical response to preventative treatment

We enrolled 22 patients (21 female, mean age 46.2 ± 13.8 years; 4 migraine with aura (MwA)) and 22 matched controls (21 female, mean age 47.6 ± 14.9 years). No patient or control was lost to follow-up, i.e. there were no drop-outs. Twelve patients received Erenumab, five patients Galcanezumab and five patients Fremanezumab monthly for an episodic migraine. Headache diaries yielded a mean headache frequency of 12.4 ± 3.3 days/month, mean duration of 8.0 ± 5.3 h/attack and mean headache intensity of 4.9 ± 1.9 at baseline. Following three months of CGRP mAb treatment, headache frequency (6.6 ± 4.9 days/month, *p*<0.001 vs. V0) and attack duration (5.4 ± 4.2 h/attack, *p*=0.014 vs. V0) significantly decreased, while headache intensity remained unchanged (5.1 ± 2.5, *p*=0.96 vs. V0). In line with headache frequency and attack duration, also MIDAS (V0: 61.7 ± 62.3, V3: 32.2 ± 61.2; *p*=0.003) and HIT-6^TM^ (V0: 66.1 ± 4.9, V3: 54.9 ± 9.2; *p*<0.001) scores improved significantly following three months of treatment. Baseline characteristics or clinical response did not differ significantly between patients who received CGRP ligand or receptor antibodies. Baseline demographic and headache characteristics were furthermore no predictors of treatment response.

### Blink reflex assessments –R2a changes in patients

**Fig. 2:**
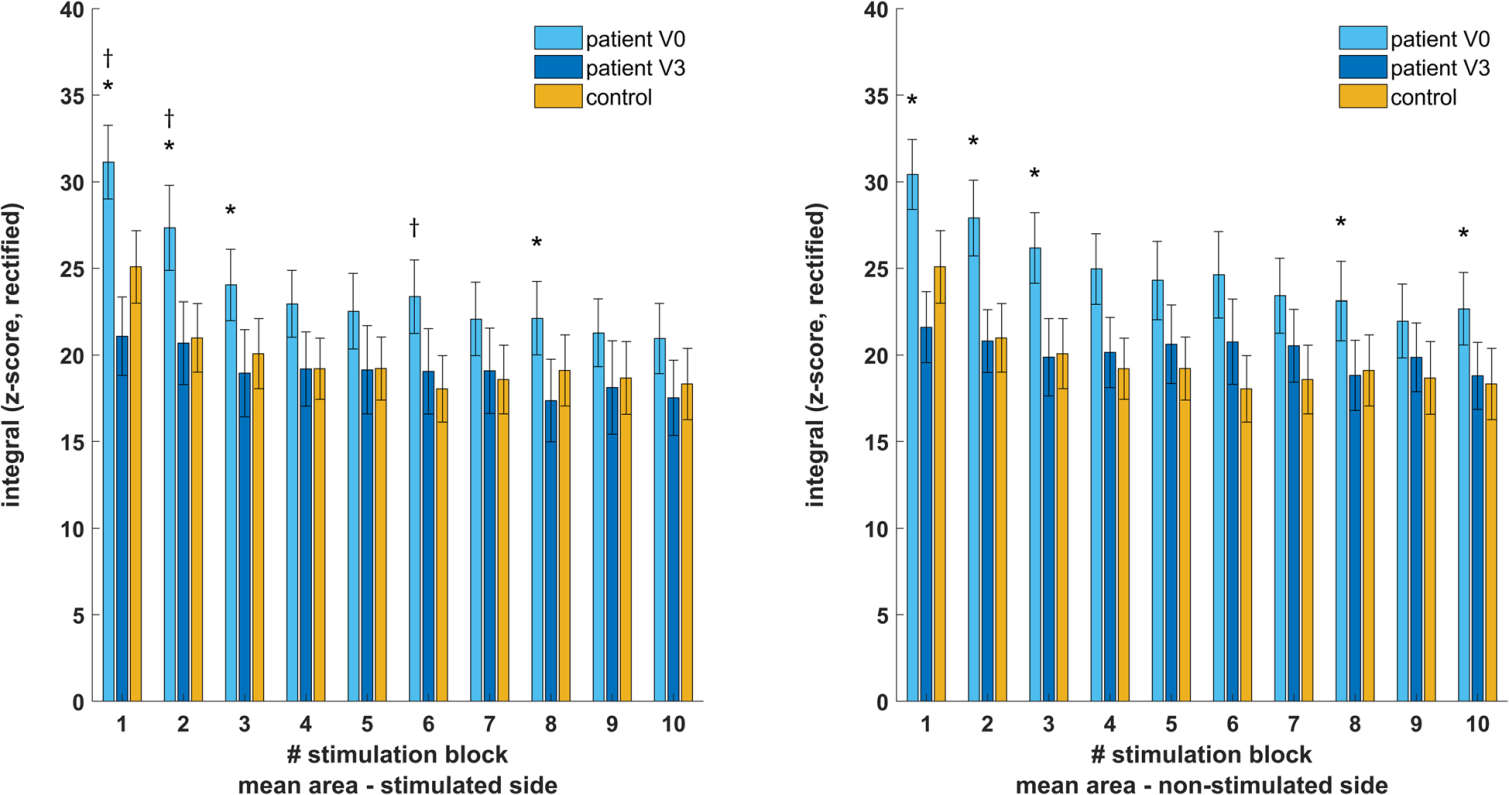
blockwise comparison of nBR excitability in patients with migraine and controls. R2a is enhanced on the stimulated and non-stimulated side in migraineurs at V0, however only changes on the stimulated were significant given the high variance. These indicators of excessive brain stem excitability normalize after three months of treatment with CGRP mAbs and are then statistically indifferent from controls. * - indicates statistically significant difference between V0 and V3 in migraine. † - indicates statistically significant difference between V0 and controls

Results of R2a changes following three months of treatment with CGRP mAbs and their comparison to healthy controls are summarized in Fig. 2. There was a significant global effect for block-wise changes of R2a_s (F_(19,597)_=5.86), *p*<0.001) and R2a_ns (F_(19,597)_=8.22), *p*<0.001) between visits. Post-hoc comparisons revealed that R2a_s significantly decreased in blocks one (-10.2 ± 2.6, *p*<0.001), two (-6.7 ± 2.8, *p*=0.028), three (-5.1 ± 2.1, *p*=0.028) and eight (-4.7 ± 2.1, *p*=0.033). R2a_ns significantly decreased in blocks one (-8.8 ± 2.6, *p*=0.003), two (-7.0 ± 2.5, *p*=0.010), three (-6.0 ± 2.5, *p*=0.025), eight (-4.4 ± 1.9, *p*=0.028) and ten (-4.1 ± 1.4, *p*=0.010).

### Blink reflex assessments – R2h changes in patients

**Fig. 3:**
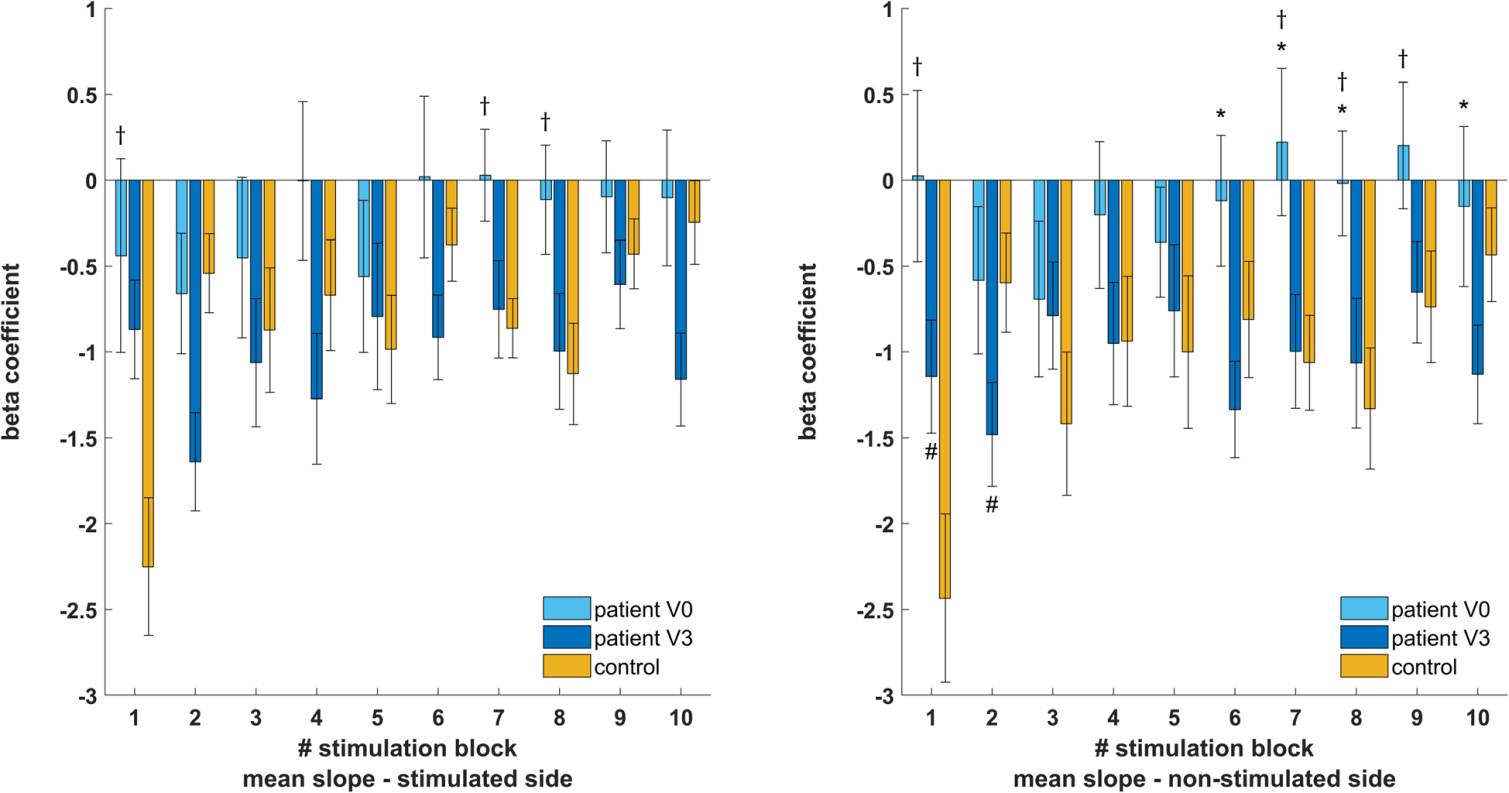
blockwise comparison of nBR habituation in patients with migraine and controls. R2h is attenuated in migraine and has even a trend towards facilitation in later blocks at V0. After three months of treatment with CGRP mAbs, R2h normalizes and consecutive stimuli are inhibited in all blocks. There remains a significant difference between V3 and controls on the non-stimulated side. * - indicates statistically significant difference between V0 and V3 in migraine. † - indicates statistically significant difference between V0 and controls. # - indicates statistically significant difference between V3 and controls

Results of R2h changes following CGRP mAb treatment and their comparison to healthy controls are summarized in Fig. 3. Global analysis for habituation only revealed significant changes on the non-stimulated side (R2h_ns; F_(19,597)_=3.07), *p*<0.001) but effects on the stimulated side (R2h_s) were marginally non-significant (F_(19,597)_=1.46, *p*=0.095). Thus, only R2h_ns effects were compared and revealed stronger attenuation of subsequent stimuli in blocks six (-1.4 ± 0.5, *p*=0.007), seven (-1.2 ± 0.4, *p*=0.010), eight (-1.0 ± 0.5, *p*=0.034) and ten (-1.0 ± 0.4, *p*=0.034) at V3 as compared to V0.

### Blink reflex assessments – comparison of patients and controls

R2a_s (F_(19,420)_=3.11, *p*<0.001), R2h_s (F_(19,420)_=2.45, *p*=0.001) and R2h_ns (F_(19,420)_=3.16, *p*<0.001) differed significantly between patients at V0 and controls, while R2a_ns was indifferent (F_(19,420)_=1.22, *p*=0.234). Neither R2a_s (F_(19,420)_=0.69, *p*=0.826), R2a_ns (F_(19,420)_=0.29, *p*=0.978) nor R2h_s (F_(19,420)_=1.38, *p*=0.195) differed between controls and patients at V3. There remained a significant global effect for R2h_ns (F_(19,420)_=2.23, *p*=0.019). For presentational purposes, comparisons to controls are not reported exact but can be found in Figs. 2 and 3.

### Electrophysiological parameters as biomarker

**Fig. 4:**
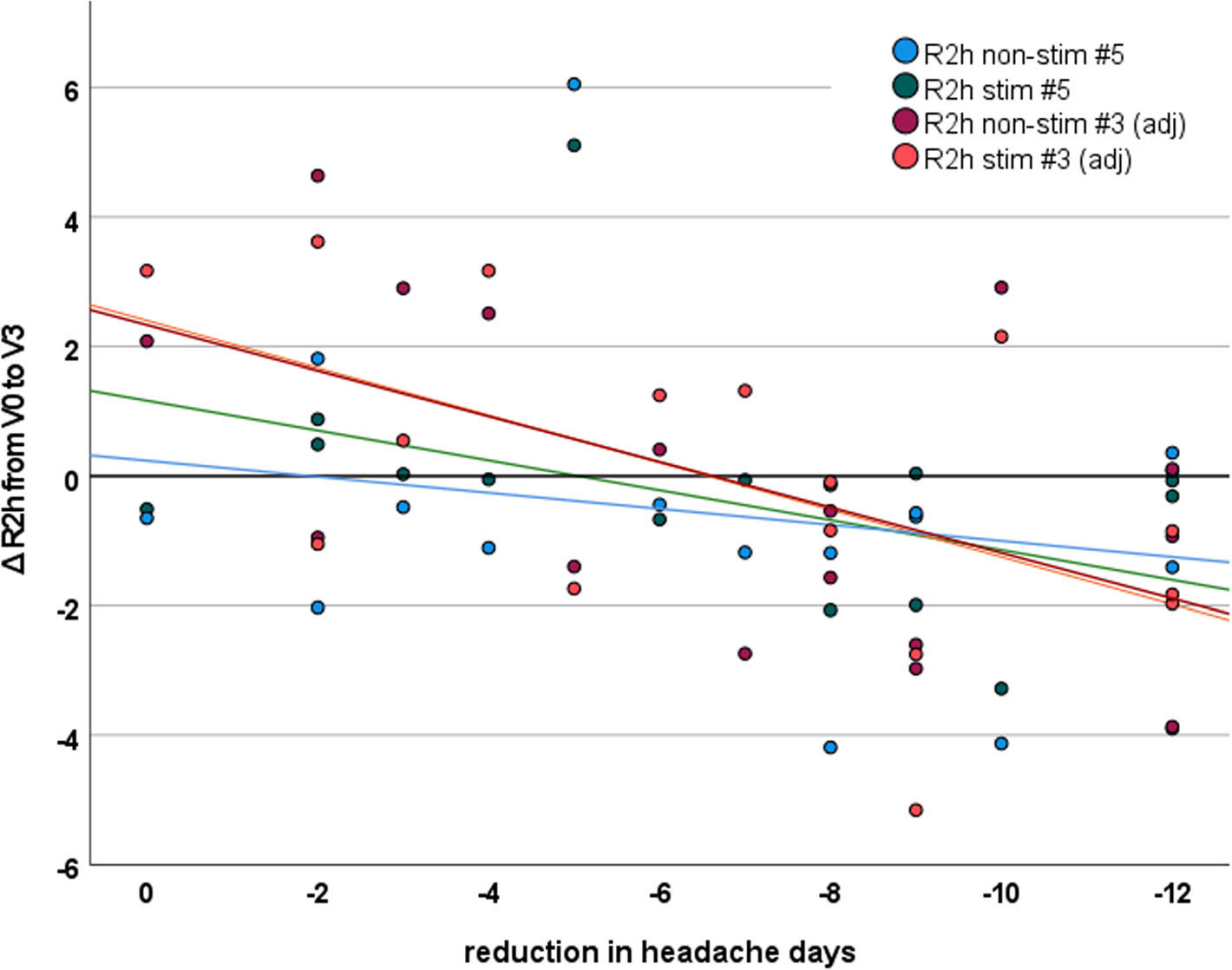
reduction in headache days as a function of R2h changes after initiation of CGRP mAbs. There is a statistically significant correlation of the change in R2h slope from V0 to V3 on the stimulated and non-stimulated side in blocks three and five. Block three results are adjusted for the time before the next headache attack. Note that the correlation coefficients reported in the results section are by definition standardized while lines plotted here are based on raw data

Changes of R2h_s (*r*=0.56, *p*=0.010) and R2h_ns (*r*=0.45, *p*=0.045) in block five significantly correlated with changes in headache frequency, while R2a on neither side was significantly correlated. When correcting for time to the next headache attack, additionally changes of R2h_s (*r*=0.56, *p*=0.025) and R2h_ns (*r*=0.54, *p*=0.030) in block three significantly correlated with improvement of headache frequency (Fig. 4).

We furthermore evaluated whether any parameter of R2a or R2h at V0 predicted treatment response. Forward stepwise linear regression revealed that neither R2a nor R2h were predictive. This was irrespective of the use of uncorrected or corrected data, and if reduction in headache days or any binary treatment response were used.

## Discussion

We were able to show that brain stem excitability and habituation to painful trigeminal stimulation are impaired in patients with migraine eligible for a course of CGRP mAbs and restored with three months of treatment. Given the significant biological gradient between normalization of nBR habituation and clinical response, treatment with CGRP mAbs can be considered causative for changes of central disease activity based on Bradford Hill criteria [34]. Since CGRP mAbs are understood to act peripherally and nBR changes are tightly associated with central disease activity, our results furthermore provide evidence that treatment with CGRP mAbs may be considered disease modifying as early as three months of treatment.

### Comparison to previous studies investigating the blink reflex as a biomarker of preventive treatment

Our literature search revealed only two studies that conducted electrophysiological investigations of the blink reflex to assess central effects of a preventive treatment. Tommaso and Delussi used the nsBR and found that treatment with Topiramate had a small effect on R2a and R2h [35]. Artemenko et al. found that duloxetine normalized the R3 threshold and habituation of the nBR [36]. However, the latter group assessed only patients with chronic migraine and it was shown that the blink reflex may not be a suitable biomarker in these patients since supraspinal structures are possibly more involved in these patients [37].

Ziegeler et al. investigated central effects following treatment with CGRP mAbs [19]. They used event-related functional magnetic resonance imaging and found a reduced hypothalamic activation in treatment responders, which supports the notion of central disease modification. Interestingly, it was shown that there is a close interaction between hypothalamic structures and the TCC in migraine pathophysiology [38]. Hence, our results are possibly a more direct and pain related read-out of central effects treatment that extend into diencephalic structures.

There are comprehensive reviews available on electrophysiological methods to investigate changes in the visual, acoustic, sensory and multimodal systems [15, 26]. It remains elusive if treatment with CGRP mAbs also improves disturbed sensory processing in these domains, which was shown for prevention with beta blockers, levetiracetam and flunarizine. Changes of sensory processing in other domains would support the notion of disease modification since migraine is considered a disorder of global sensory processing [20].

### Comparison of the nociception specific and nociceptive stimulation

There is a large heterogeneity in the literature considering the use of either the nBR or nsBR in the investigation of migraine pathophysiology. Pharmacological studies have shed light on divergent and shared pathways between both techniques and need to be considered. Migraine-associated changes of the nsBR were found to be restored by treatment with triptanes in two studies [39, 40]. Another study compared the nBR and nsBR using an adenosine receptor agonist, which is considered to inhibit release of neuropeptides on terminal trigeminal nerve fibers including CGRP. This study found that the nsBR but not nBR was changed by application of the agonist [29]. Thus, current understanding is that the nsBR and nBR share common central pathways involving the TCC and reflecting migraine pathophysiology, but the afferent input differs between both variants. The nsBR is largely mediated by Aδ-fibers and the nBR mainly depends on C-fiber activation, which renders the nsBR more susceptible to peripheral CGRP-mediated effects on trigeminal afferent pathways than the nBR. Assessments of the nBR are thus more robust against confounding effects of CGRP mAbs on a peripheral level and better suited to assess central effects in the TCC .

### Utility of the nBR as biomarker in clinical practice

We found that changes of R2h correlated with changes of headache frequency. Hence, the nBR can be considered a biomarker by definition of the FDA Biomarker Working Group [41]. While we were able to provide evidence for the nBR as a biomarker to monitor treatment response, our results unfortunately did not reveal any parameter that predicts the treatment response at baseline. Following the evidence that the nsBR is possibly more specific for CGRP-dependent activation of the TCC, it might be better suited to predict treatment response and less suitable for monitoring the treatment (please also see discussion in the previous paragraph). It remains unclear at present if the nBR as monitoring biomarker can predict the risk of relapse after discontinuation of the preventive drug. This would be of significant clinical value since clinical response fades in about one third of patients as early as one month after discontinuation of treatment with CGRP mAbs, less than every second patient sustains a meaningful ≥30% or ≥50% reduction in headache days and most patients consecutively restart treatment after three months [42–44]. Future studies should thus investigate changes of the baseline nBR during treatment, before and after discontinuation to assess its predictive value. If the nBR was a suitable biomarker, treatment duration might be tailored to individual needs.

### Are CGRP mAbs disease modifying?

In the introduction, we summarized that a DMMD should slow down the natural course of the disease. We indeed found that a course of CGRP mAbs not only leads to a clinical response through peripheral antagonism of migraine attacks but restores the brainstem nociceptive system, which is in line with the EMA’s definition of disease modification in neurodegenerative disease [13]. On a clinical level, Lipton et al. showed that CGRP mAbs can lead to a reversion from chronic to episodic migraine, which further supports the notion of CGRP mAbs as DMMD since the natural course of episodic migraine is to convert to chronic migraine at a rate of about 5%/year [45]. On the other hand, there is a lack of sustained reduction of headache frequency after discontinuation of treatment, which apparently contradicts disease modification [42–45]. Our study supports the notion of CGRP mAbs as DMMD but future studies need to assess if biomarker changes of central disease activity are sustained in the discontinuation period.

## Limitations

We investigated only episodic migraine since at least a proportion of patients with chronic migraine is known to lack a clear interictal phase that impedes electrophysiological assessments outside a migraine attack [25]. Nonetheless, central disease modifying activity should not differ between episodic and chronic migraine since both are known to be ameliorated by CGRP mAbs [3]. Future studies, however, that investigate any variant of the blink reflex as clinical biomarker should include patients with chronic migraine since their disease burden is particularly high and they are more likely to receive a treatment with CGRP mAbs [7, 46]. Subsequent studies should also include repeated measurements of control subjects, since there might be an adaptation or natural fluctuation of the nBR response. This approach would enable a more precise estimate of the sole effect caused by CGRP mAbs. The direction of findings from this study, however, would remain unchanged since we already found a non-physiological nBR response in patients with migraine at baseline, which is not to be expected in controls based on multiple studies using the same protocol [23, 26].

Another limitation is that we cannot exclude non-pharmacological effects on central disease activity. Our patients revealed a treatment response that is in line with what is to be expected, which improved their ability to pursue non-pharmacological interventions such as physical activity and relaxation techniques [47]. Unfortunately, we did not record these interventions but in a study that investigated the nsBR in the context of biofeedback, the authors did not find a change of habituation, which we found to correlate with pharmacological treatment response [35].

Any variant of the blink reflex can be elicited at different interstimulus intervals with shorter intervals causing more habituation [23]. We used an interstimulus interval that was most often used in previous studies and thus enables comparisons to previous studies. We cannot rule out the possibility that shorter interstimulus intervals would have led to different results. Future studies should thus include a set of discrete time intervals.

## Conclusions

The correlation of changes of the nBR habituation with clinical response provides evidence for disease modifying potential of three months of treatment with CGRP mAbs. Our findings render the nBR habituation a potential biomarker to monitor treatment response in clinical practice. Unfortunately, we were unable to find that any nBR parameter predicted treatment response. Future studies should evaluate both variants of the BR, i.e. the nBR and nsBR, for their utility as clinical biomarkers to predict treatment response at baseline and to establish the risk of relapse after treatment discontinuation.

## Data Availability

The datasets generated and/or analysed during the current study are not publicly available due to data protection regulations that impede distribution but are available from the corresponding author on reasonable request.
